# Gangrene of the penis in a diabetic male with multiple amputations and follow up

**DOI:** 10.4103/0970-1591.45550

**Published:** 2009

**Authors:** P. Vijayan

**Affiliations:** Department of Urology, St Philomena's Hospital, Mother Theresa Road, Bangalore-560 047, India

**Keywords:** Amputation, diabetic, gangrene, penis

## Abstract

A 60-year-old insulin dependent, diabetic male with severe atherosclerosis requiring multiple amputations in the extremities in the past, with normal renal function presented with gangrene of glans penis. He was initially treated with debridement but as the gangrene progressed, partial penile amputation was performed. He showed no further progress of the disease and had no voiding difficulties even after 4 years of follow up.

## INTRODUCTION

Penile gangrene is rare and can pose difficulties in management.[1] Dry gangrene is usually the end result of vascular compromise. Although the distal penis and glans have plentiful arterial supply, arterial occlusion can cause distal necrosis similar to ischaemic gangrene often noted in the digits of extremities.[2] There have been in the urological literature, sporadic reports of solitary cases of penile gangrene and a retrospective study of seven cases associated with diabetes mellitus and about 34 cases associated with chronic renal failure on hemodialysis.[3,4] Diabetes mellitus, end stage renal disease or tourniquet syndrome, may cause progressive vaso-occlusive changes. Patients with diabetes and chronic renal failure often have histories of hospitalization related to systemic effects of vascular disease. Many are ill though not in terminal stage. The other causes of penile gangrene are penile prosthesis, tourniquet effect created by condom appliances, thrombo-embolic phenomena, and hyper-coagulopathy secondary to neoplastic diseases.[5]

## CASE REPORT

On 12/8/03, a 60-year-old diabetic male, presented with necrosis of the tip of glans penis of 1week duration. He had undergone transuretheral prostatectomy (TURP), 4 months earlier for bladder outlet obstructive symptoms at another hospital. He developed severe bladder neck contracture resulting in retention of urine 1 month after the procedure and required suprapubic cystostomy.

Earlier, patient had undergone left above knee (1998) right below knee (2001) and amputation of 2 fingers of the left hand (1999).

**Figure 1:**
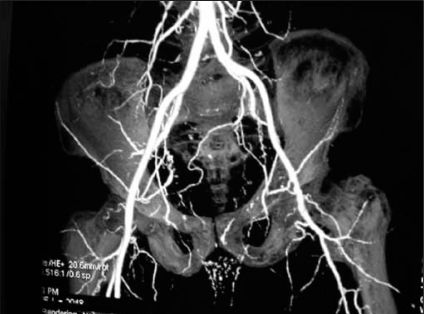
CT angiogram showing multiple areas of stenosis and occlusion in branches of internal iliac arteris bilaterally

On examination, there was a dry, dark gangrenous patch 1×1.5 cm over the glans penis at the external urinary meatus. A suprapubic catheter was in situ. His diabetes was well controlled with insulin. Blood investigations showed Hb 13.5g/dL, fasting blood sugar 80mg/dL, post prandial blood sugar 210 mg/dL, urea 41mg/dL, and creatinine 1.4mg/dL. The values for serum calcium and phosphorus were 8.8 mg/dL and 3mg/dL, respectively. The parathormone was markedly raised at 425-pg/ml. 2D echocardiogram revealed mild left ventricular dysfunction. Computed tomography angiogram showed severe atherosclerotic vascular disease resulting in multiple areas of stenosis and occlusion in branches internal iliac arteries bilaterally. The superficial femoral artery on the left side was completely occluded and on the right, the arterial wall showed foci of calcification, irregularity and distally about 20% narrowing [Figure 1].

5 days after admission, under general anaesthesia, the gangrenous patch on the glans penis was excised. After exposing the distal urethra, urethroscopy showed almost complete obliteration of bladder neck allowing passage of only a guide wire. Multiple radiate bladder neck incisions were made with a cold knife. A urethral catheter was left in position. He was discharged home few days later.

**Figure 2:**
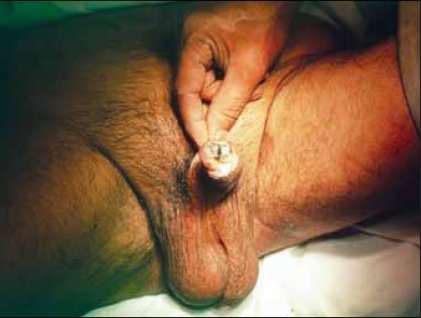
Gangrenous tip at the glans penis

He was readmitted one week later, with further gangrene involving the distal half of glans penis with some discharge. A partial amputation was carried out [Figure 2]. The urethra was brought out through the ventral flap. Following removal of catheter 1 week later, he was voiding well and the wound had healed well. He was regularly followed up quarterly. Histopathology of the initially debrided tissue revealed gangrenous change in the mucosa and deeper tissue. The amputated specimen showed extensive necrosis and scanty infiltrate at the junction with the viable tissue. An artery with organized thrombus was also seen. The gangrene probably resulted from thrombosis of an “end artery” in the glans penis.

**Figure 3:**
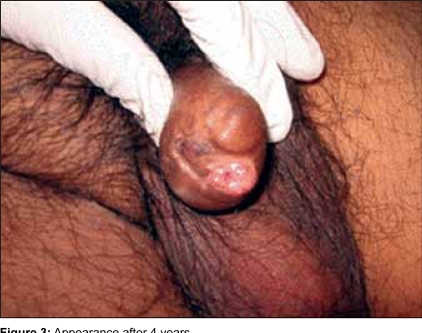
Appearance after 4 years

He was last reviewed more than 4 years after the operation. He had no voiding difficulties. The amputated penile stump was looking good. The urethra was pink and healthy [Figure 3].

## DISCUSSION

Penile gangrene is a hallmark of severe systemic vascular disease. Weiner *et al.* reported 57% mortality within 6 months of presentation. It is not surprising that these diabetics with penile gangrene have high mortality rate.[4] However Stein *et al.* concluded that surgical treatment of ischemic penile gangrene associated with chronic renal failure suffered 71% mortality and hence offered no surgical advantage over observation alone.[3]

Weiner *et al.*[4] also noted in their series that observation initially led to liquefaction as opposed to mummification and auto-amputation. All the four patients treated conservatively had progression from dry to wet gangrene within a short time. Early surgical management of penile gangrene can improve quality of life of the patient in regard to wound care and lower urinary tract management. Despite high mortality rate associated with ischaemic penile gangrene in diabetic patients with or without renal failure, aggressive management is recommended for those who are not terminal or moribund.

The index case was neither in renal failure nor on hemodialysis. The product [Ca] X [PO4] was (8.8 X 3) 26.4mg^2^/dL^2^ and well within the normal range (20.6–52.5 mg). Though the parathormone level was raised, there was no evidence of calciphiilaxis. Angiographic studies did reveal evidence of severe atherosclerosis particularly affecting the branches of internal iliac and superficial femoral arteries more pronounced on the left side. It is difficult to conclude that this could have caused pelvic ischaemia leading to bladder neck contracture.

In the presence of such severe atherosclerosis in an elderly diabetic male, it is doubtful if re-vascularizing procedures would succeed.

With appropriate patient selection, surgical intervention can be successful and provide a better quality of life for those without terminal disease as illustrated by the above case who has now been followed up for 4 years since the event.

## References

[1] Harris FC, Mydlo JH (2003). Ischaemia and gangrene of penis. J Urol.

[2] Gillitzer R, Franzaring L, Hampel C, Pahernik S, Bittinger F, Thüroff JW (2004). Complete gangrene of penis in a patient with arterial vascular disease. Urology.

[3] Stein M, Anderson C, Ricciardi R, Chamberlain JW, Lerner SE, Glicklich D (1994). Penile gangrene: Associated with chronic renal failure: Report of 7 cases and review of literature. J Urol.

[4] Buyukasik Y, sayinalp N, Anci M, Haznedaroglu IC, Dundar S (1997). Penile gangrene in lung cancer. Postgrad Med J.

[5] Weiner DM, Lowe FC (1996). Surgical management of ischaemic penile gangrene in diabetics with end stage atherosclerosis. J Urol.

